# 1-(5-Bromo-2-chloro­phen­yl)-2,2-di­chloro-1-(4-eth­oxy­phen­yl)cyclo­propane

**DOI:** 10.1107/S1600536813003735

**Published:** 2013-02-20

**Authors:** Shuwen Han, Yongheng Shi, Wang Yuli, Guilong Zhao, Weiren Xu

**Affiliations:** a Graduate School, Tianjin University of Traditional Chinese Medicine, Tianjin 300193, People’s Republic of China; b Tianjin Key Laboratory of Molecular Design and Drug Discovery, Tianjin Institute of Pharmaceutical Research, Tianjin 300193, People’s Republic of China

## Abstract

The asymmetric unit of the title compound, C_17_H_14_BrCl_3_O, contains two independent mol­ecules with different dihedral angles between the benzene rings [79.2 (1) and 72.7 (1)°]. In the crystal, weak C—H⋯π inter­actions link mol­ecules related by translation along the *b* axis into two crystallographically independent chains.

## Related literature

For background to sodium-glucose cotransporter 2 (SGLT2) inhibitors, see: Washburn (2009 ▶); Meng *et al.* (2008 ▶). For the crystal structures of related cyclo­propane derivatives, see: DeLacy & Kennard (1972 ▶); Lauher & Ibers (1975 ▶).
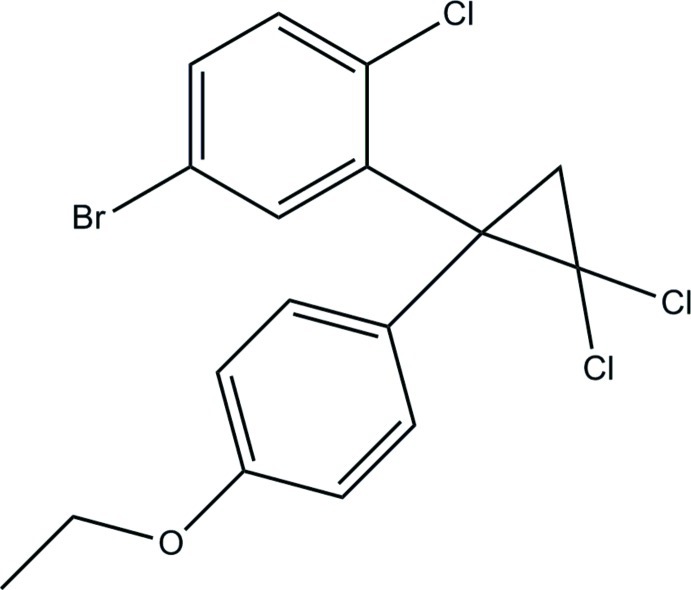


## Experimental

### 

#### Crystal data

C_17_H_14_BrCl_3_O
*M* *_r_* = 420.54
Monoclinic, 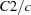

*a* = 27.447 (13) Å
*b* = 8.886 (4) Å
*c* = 28.861 (14) Å
β = 98.348 (8)°
*V* = 6965 (6) Å^3^
*Z* = 16
Mo *K*α radiation
μ = 2.82 mm^−1^
*T* = 113 K
0.20 × 0.18 × 0.14 mm

#### Data collection

Rigaku Saturn724 CCD diffractometer
Absorption correction: multi-scan (*CrystalClear*; Rigaku/MSC, 2009 ▶) *T*_min_ = 0.603, *T*_max_ = 0.694
26327 measured reflections
6143 independent reflections
4903 reflections with *I* > 2σ(*I*)
*R*_int_ = 0.104

#### Refinement

*R*[*F* ^2^ > 2σ(*F* ^2^)] = 0.067
*wR*(*F* ^2^) = 0.180
*S* = 1.09
6143 reflections
399 parameters
H-atom parameters constrained
Δρ_max_ = 1.54 e Å^−3^
Δρ_min_ = −0.89 e Å^−3^

### 

Data collection: *CrystalClear* (Rigaku/MSC, 2009 ▶); cell refinement: *CrystalClear*; data reduction: *CrystalClear*; program(s) used to solve structure: *SHELXS97* (Sheldrick, 2008 ▶); program(s) used to refine structure: *SHELXL97* (Sheldrick, 2008 ▶); molecular graphics: *SHELXTL* (Sheldrick, 2008 ▶); software used to prepare material for publication: *SHELXTL*.

## Supplementary Material

Crystal structure: contains datablock(s) I, global. DOI: 10.1107/S1600536813003735/cv5386sup1.cif

Structure factors: contains datablock(s) I. DOI: 10.1107/S1600536813003735/cv5386Isup2.hkl

Supplementary material file. DOI: 10.1107/S1600536813003735/cv5386Isup3.cml

Additional supplementary materials:  crystallographic information; 3D view; checkCIF report

## Figures and Tables

**Table 1:** Hydrogen-bond geometry (Å, °) *Cg*1 is the centroid of the C10–C15 ring and *Cg*2 is the centroid of the C27–C32 ring.

*D*—H⋯*A*	*D*—H	H⋯*A*	*D*⋯*A*	*D*—H⋯*A*
C3—H3⋯*Cg*1^i^	0.95	2.66	3.665 (3)	161
C20—H20⋯*Cg*2^ii^	0.95	2.60	3.480 (9)	155

Symmetry codes: (i) 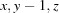; (ii) 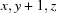.

**Fig. 1.**
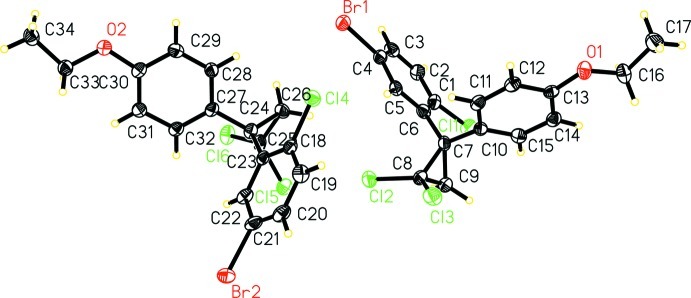
View of the title compound showing the atomic numbering and 40% probability displacement ellipsoids.

## supplementary crystallographic information

### Comment

Sodium-glucose cotransporter 2 (SGLT2) inhibitors are a new class of anti-diabetic drugs with novel mechanism of action. The most advanced drug, dapagliflozin, has recently been approved in EU (Meng *et al.*, 2008; Washburn, 2009). During our search for new SGLT2 inhibitors, we prepared the title compound as a key intermediate for the synthesis of a new class of cyclopropane-bearing SGLT2 inhibitors.

The asymmetric unit of the title compound, C_17_H_14_BrCl_3_O, contains two independent molecules with different dihedral angles between the benzene rings [79.2 (1) and 72.7 (1)°]. Bond lengths are normal and in a good agreement with those reported previously for related structures (DeLacy & Kennard, 1972; Lauher & Ibers 1975). In the crystal, weak C—H···π interactions (Table 1) link the molecules related by translation along the axis *b* into two crystallographically independent chains.

### Experimental

3.38 g (10 mmol) of 1-(5-bromo-2-chlorophenyl)-1-(4-ethoxyphenyl)ethene was dissolved in 15 ml of chloroform and stirred at room temperature, followed by addition of 3 ml of 50% aqueous NaOH and 0.5 g of benzyltriethylammonium bromide. The reaction mixture was stirred vigorously at room temperature overnight until all the starting ethene was consumed as indicated by TLC. The reaction mixture was poured into 200 ml of water and extracted with three 50-ml portions of dichloromethane. The combined extracts were washed with saturated brine, dried over anhydrous sodium sulfate and evaporated on a rotary evaporator to afford the crude product as a colorless oil, which was purified by column chromatography to yield the pure product as colorless crystals. Single crystals suitable for X-ray diffraction were obtained through slow evaporation of a solution of the pure title compound in petroleum ether.

### Refinement

All H atoms were geometrically positioned, with C–H = 0.95–0.99 Å, and included in the final cycles of refinement using a riding model, with *U*_iso_(H) = 1.2–1.5 *U*_eq_(C).

### Crystal data

**Table:**

C_17_H_14_BrCl_3_O	*F*(000) = 3360
*M**_r_* = 420.54	*D*_x_ = 1.604 Mg m^−^^3^
Monoclinic, *C*2/*c*	Mo *K*α radiation, λ = 0.71073 Å
Hall symbol: -C 2yc	Cell parameters from 11664 reflections
*a* = 27.447 (13) Å	θ = 1.5–28.0°
*b* = 8.886 (4) Å	µ = 2.82 mm^−^^1^
*c* = 28.861 (14) Å	*T* = 113 K
β = 98.348 (8)°	Prism, colorless
*V* = 6965 (6) Å^3^	0.20 × 0.18 × 0.14 mm
*Z* = 16	

### Data collection

**Table:**

Rigaku Saturn724 CCD diffractometer	6143 independent reflections
Radiation source: rotating anode	4903 reflections with *I* > 2σ(*I*)
Multilayer monochromator	*R*_int_ = 0.104
Detector resolution: 14.22 pixels mm^-1^	θ_max_ = 25.0°, θ_min_ = 1.5°
ω and φ scans	*h* = −32→32
Absorption correction: multi-scan (*CrystalClear*; Rigaku/MSC, 2009)	*k* = −10→10
*T*_min_ = 0.603, *T*_max_ = 0.694	*l* = −34→33
26327 measured reflections	

### Refinement

**Table:**

Refinement on *F*^2^	Primary atom site location: structure-invariant direct methods
Least-squares matrix: full	Secondary atom site location: difference Fourier map
*R*[*F*^2^ > 2σ(*F*^2^)] = 0.067	Hydrogen site location: inferred from neighbouring sites
*wR*(*F*^2^) = 0.180	H-atom parameters constrained
*S* = 1.09	*w* = 1/[σ^2^(*F*_o_^2^) + (0.0747*P*)^2^] where *P* = (*F*_o_^2^ + 2*F*_c_^2^)/3
6143 reflections	(Δ/σ)_max_ = 0.003
399 parameters	Δρ_max_ = 1.54 e Å^−^^3^
0 restraints	Δρ_min_ = −0.89 e Å^−^^3^

### Special details

**Table:**

Geometry. All e.s.d.'s (except the e.s.d. in the dihedral angle between two l.s. planes) are estimated using the full covariance matrix. The cell e.s.d.'s are taken into account individually in the estimation of e.s.d.'s in distances, angles and torsion angles; correlations between e.s.d.'s in cell parameters are only used when they are defined by crystal symmetry. An approximate (isotropic) treatment of cell e.s.d.'s is used for estimating e.s.d.'s involving l.s. planes.
Refinement. Refinement of *F*^2^ against ALL reflections. The weighted *R*-factor *wR* and goodness of fit *S* are based on *F*^2^, conventional *R*-factors *R* are based on *F*, with *F* set to zero for negative *F*^2^. The threshold expression of *F*^2^ > σ(*F*^2^) is used only for calculating *R*-factors(gt) *etc*. and is not relevant to the choice of reflections for refinement. *R*-factors based on *F*^2^ are statistically about twice as large as those based on *F*, and *R*- factors based on ALL data will be even larger.

### Fractional atomic coordinates and isotropic or equivalent isotropic displacement parameters (Å^2^)

**Table:**

	*x*	*y*	*z*	*U*_iso_*/*U*_eq_	
Br1	0.67589 (2)	−0.00608 (7)	0.06050 (2)	0.0372 (2)	
Br2	0.43550 (2)	0.53077 (7)	0.20897 (2)	0.0428 (2)	
Cl1	0.83567 (5)	0.28486 (18)	0.22003 (5)	0.0403 (4)	
Cl2	0.65376 (5)	0.41196 (18)	0.19345 (5)	0.0439 (4)	
Cl3	0.67918 (6)	0.70547 (18)	0.15844 (6)	0.0478 (4)	
Cl4	0.58075 (5)	0.28004 (17)	0.07179 (5)	0.0386 (4)	
Cl5	0.58547 (5)	0.14483 (17)	0.24050 (5)	0.0392 (4)	
Cl6	0.55111 (5)	−0.15899 (17)	0.21433 (5)	0.0427 (4)	
O1	0.85763 (13)	0.7592 (4)	0.03892 (12)	0.0354 (9)	
O2	0.38664 (14)	−0.2563 (5)	0.03844 (13)	0.0373 (10)	
C1	0.7900 (2)	0.2085 (7)	0.17851 (18)	0.0329 (13)	
C2	0.7932 (2)	0.0558 (7)	0.1666 (2)	0.0361 (14)	
H2	0.8188	−0.0044	0.1828	0.043*	
C3	0.7602 (2)	−0.0070 (6)	0.1322 (2)	0.0356 (14)	
H3	0.7634	−0.1093	0.1236	0.043*	
C4	0.7210 (2)	0.0825 (6)	0.10951 (18)	0.0321 (13)	
C5	0.71635 (19)	0.2331 (6)	0.12209 (19)	0.0308 (12)	
H5	0.6896	0.2911	0.1070	0.037*	
C6	0.7505 (2)	0.2992 (6)	0.15645 (19)	0.0321 (13)	
C7	0.74841 (19)	0.4669 (6)	0.1662 (2)	0.0322 (13)	
C8	0.7021 (2)	0.5333 (6)	0.1829 (2)	0.0369 (14)	
C9	0.7478 (2)	0.5258 (7)	0.2166 (2)	0.0377 (14)	
H9A	0.7494	0.4505	0.2421	0.045*	
H9B	0.7651	0.6215	0.2256	0.045*	
C10	0.77570 (19)	0.5583 (6)	0.13452 (19)	0.0295 (12)	
C11	0.7582 (2)	0.5712 (6)	0.08606 (19)	0.0321 (13)	
H11	0.7268	0.5310	0.0742	0.038*	
C12	0.7856 (2)	0.6408 (6)	0.05556 (19)	0.0328 (13)	
H12	0.7727	0.6478	0.0233	0.039*	
C13	0.83215 (19)	0.7009 (6)	0.07179 (18)	0.0277 (12)	
C14	0.8501 (2)	0.6955 (6)	0.12017 (19)	0.0330 (13)	
H14	0.8807	0.7411	0.1321	0.040*	
C15	0.82230 (19)	0.6219 (6)	0.15032 (19)	0.0315 (13)	
H15	0.8353	0.6145	0.1826	0.038*	
C16	0.9085 (2)	0.8066 (7)	0.0533 (2)	0.0426 (15)	
H16A	0.9278	0.7234	0.0697	0.051*	
H16B	0.9096	0.8936	0.0748	0.051*	
C17	0.9298 (3)	0.8496 (8)	0.0099 (2)	0.0550 (18)	
H17A	0.9298	0.7617	−0.0106	0.082*	
H17B	0.9637	0.8854	0.0188	0.082*	
H17C	0.9099	0.9298	−0.0067	0.082*	
C18	0.54406 (19)	0.3437 (6)	0.11248 (17)	0.0292 (12)	
C19	0.5307 (2)	0.4958 (7)	0.1094 (2)	0.0367 (14)	
H19	0.5434	0.5598	0.0876	0.044*	
C20	0.4985 (2)	0.5532 (7)	0.1387 (2)	0.0376 (14)	
H20	0.4886	0.6558	0.1368	0.045*	
C21	0.4812 (2)	0.4555 (6)	0.1706 (2)	0.0367 (14)	
C22	0.49516 (19)	0.3061 (6)	0.17397 (18)	0.0310 (13)	
H22	0.4828	0.2432	0.1962	0.037*	
C23	0.52744 (18)	0.2449 (6)	0.14489 (17)	0.0265 (12)	
C24	0.53880 (19)	0.0807 (6)	0.14758 (18)	0.0293 (12)	
C25	0.5670 (2)	0.0172 (6)	0.1931 (2)	0.0338 (13)	
C26	0.5933 (2)	0.0272 (7)	0.1520 (2)	0.0357 (14)	
H26A	0.6000	−0.0677	0.1361	0.043*	
H26B	0.6191	0.1052	0.1524	0.043*	
C27	0.4984 (2)	−0.0153 (6)	0.12023 (18)	0.0263 (12)	
C28	0.50266 (19)	−0.0656 (6)	0.07508 (18)	0.0300 (12)	
H28	0.5320	−0.0454	0.0623	0.036*	
C29	0.4652 (2)	−0.1437 (6)	0.04900 (19)	0.0332 (13)	
H29	0.4687	−0.1757	0.0182	0.040*	
C30	0.42187 (19)	−0.1767 (6)	0.06726 (17)	0.0283 (12)	
C31	0.4166 (2)	−0.1279 (6)	0.11244 (18)	0.0297 (12)	
H31	0.3874	−0.1492	0.1252	0.036*	
C32	0.45541 (19)	−0.0464 (6)	0.13867 (19)	0.0298 (12)	
H32	0.4521	−0.0125	0.1693	0.036*	
C33	0.3404 (2)	−0.2861 (7)	0.05542 (19)	0.0340 (13)	
H33A	0.3243	−0.1905	0.0621	0.041*	
H33B	0.3461	−0.3464	0.0845	0.041*	
C34	0.3086 (2)	−0.3721 (7)	0.0173 (2)	0.0386 (14)	
H34A	0.3027	−0.3104	−0.0111	0.058*	
H34B	0.2770	−0.3965	0.0276	0.058*	
H34C	0.3253	−0.4654	0.0106	0.058*	

### Atomic displacement parameters (Å^2^)

**Table:**

	*U*^11^	*U*^22^	*U*^33^	*U*^12^	*U*^13^	*U*^23^
Br1	0.0356 (4)	0.0322 (4)	0.0441 (4)	−0.0030 (3)	0.0068 (3)	−0.0046 (3)
Br2	0.0407 (4)	0.0353 (4)	0.0537 (4)	0.0065 (3)	0.0111 (3)	−0.0098 (3)
Cl1	0.0385 (8)	0.0353 (9)	0.0449 (9)	0.0014 (6)	−0.0016 (6)	−0.0038 (6)
Cl2	0.0396 (8)	0.0374 (9)	0.0579 (10)	−0.0057 (7)	0.0182 (7)	−0.0038 (7)
Cl3	0.0488 (9)	0.0329 (9)	0.0652 (10)	0.0102 (7)	0.0195 (7)	0.0036 (7)
Cl4	0.0384 (8)	0.0356 (9)	0.0433 (8)	0.0013 (6)	0.0107 (6)	−0.0006 (6)
Cl5	0.0407 (8)	0.0373 (9)	0.0379 (8)	−0.0026 (6)	−0.0002 (6)	0.0011 (6)
Cl6	0.0409 (8)	0.0288 (8)	0.0557 (9)	−0.0014 (6)	−0.0019 (7)	0.0141 (7)
O1	0.035 (2)	0.034 (2)	0.036 (2)	−0.0050 (18)	0.0020 (17)	0.0074 (17)
O2	0.035 (2)	0.041 (3)	0.037 (2)	−0.0066 (18)	0.0090 (17)	−0.0073 (18)
C1	0.031 (3)	0.035 (4)	0.031 (3)	−0.005 (3)	0.001 (2)	0.003 (2)
C2	0.030 (3)	0.028 (3)	0.049 (4)	0.006 (3)	0.005 (3)	0.002 (3)
C3	0.037 (3)	0.026 (3)	0.048 (4)	0.007 (3)	0.017 (3)	0.003 (3)
C4	0.034 (3)	0.025 (3)	0.039 (3)	−0.002 (2)	0.010 (2)	0.000 (2)
C5	0.027 (3)	0.025 (3)	0.042 (3)	0.000 (2)	0.011 (2)	0.003 (2)
C6	0.035 (3)	0.027 (3)	0.037 (3)	0.001 (2)	0.013 (2)	0.004 (2)
C7	0.026 (3)	0.024 (3)	0.048 (4)	−0.003 (2)	0.012 (2)	−0.002 (2)
C8	0.042 (3)	0.020 (3)	0.052 (4)	−0.004 (3)	0.017 (3)	−0.005 (3)
C9	0.039 (3)	0.031 (4)	0.044 (4)	−0.006 (3)	0.012 (3)	−0.005 (3)
C10	0.026 (3)	0.024 (3)	0.039 (3)	0.001 (2)	0.008 (2)	−0.005 (2)
C11	0.031 (3)	0.026 (3)	0.038 (3)	0.001 (2)	0.000 (2)	−0.003 (2)
C12	0.036 (3)	0.027 (3)	0.034 (3)	0.002 (2)	0.001 (2)	−0.003 (2)
C13	0.033 (3)	0.020 (3)	0.029 (3)	0.004 (2)	0.005 (2)	−0.002 (2)
C14	0.034 (3)	0.025 (3)	0.040 (3)	0.001 (3)	0.004 (2)	−0.001 (2)
C15	0.030 (3)	0.030 (3)	0.034 (3)	0.008 (2)	0.003 (2)	0.002 (2)
C16	0.032 (3)	0.045 (4)	0.052 (4)	−0.004 (3)	0.010 (3)	0.005 (3)
C17	0.059 (4)	0.051 (5)	0.061 (4)	0.001 (4)	0.026 (3)	0.005 (3)
C18	0.031 (3)	0.028 (3)	0.028 (3)	−0.005 (2)	0.002 (2)	−0.003 (2)
C19	0.039 (3)	0.034 (4)	0.037 (3)	0.001 (3)	0.004 (3)	0.008 (3)
C20	0.046 (4)	0.021 (3)	0.043 (4)	0.002 (3)	−0.002 (3)	−0.001 (3)
C21	0.040 (3)	0.024 (3)	0.043 (3)	0.006 (3)	−0.002 (3)	−0.010 (3)
C22	0.034 (3)	0.021 (3)	0.034 (3)	0.006 (2)	−0.007 (2)	0.000 (2)
C23	0.021 (3)	0.025 (3)	0.031 (3)	0.000 (2)	−0.003 (2)	−0.003 (2)
C24	0.026 (3)	0.022 (3)	0.038 (3)	0.002 (2)	−0.001 (2)	0.002 (2)
C25	0.028 (3)	0.024 (3)	0.048 (4)	−0.004 (2)	0.002 (3)	0.005 (3)
C26	0.028 (3)	0.033 (4)	0.047 (4)	0.005 (3)	0.009 (3)	−0.003 (3)
C27	0.031 (3)	0.014 (3)	0.034 (3)	0.001 (2)	0.002 (2)	0.001 (2)
C28	0.026 (3)	0.031 (3)	0.033 (3)	0.000 (2)	0.005 (2)	−0.001 (2)
C29	0.035 (3)	0.033 (3)	0.033 (3)	0.002 (3)	0.009 (2)	−0.001 (2)
C30	0.034 (3)	0.018 (3)	0.032 (3)	0.001 (2)	0.000 (2)	0.003 (2)
C31	0.033 (3)	0.020 (3)	0.037 (3)	0.001 (2)	0.007 (2)	0.002 (2)
C32	0.034 (3)	0.021 (3)	0.034 (3)	0.002 (2)	0.002 (2)	−0.001 (2)
C33	0.033 (3)	0.031 (3)	0.039 (3)	−0.002 (3)	0.006 (2)	−0.001 (2)
C34	0.034 (3)	0.030 (4)	0.053 (4)	−0.001 (3)	0.011 (3)	0.001 (3)

### Geometric parameters (Å, º)

**Table:**

Br1—C4	1.908 (5)	C16—C17	1.505 (8)
Br2—C21	1.912 (6)	C16—H16A	0.9900
Cl1—C1	1.742 (5)	C16—H16B	0.9900
Cl2—C8	1.770 (6)	C17—H17A	0.9800
Cl3—C8	1.763 (6)	C17—H17B	0.9800
Cl4—C18	1.748 (6)	C17—H17C	0.9800
Cl5—C25	1.793 (6)	C18—C19	1.400 (8)
Cl6—C25	1.759 (6)	C18—C23	1.406 (7)
O1—C13	1.360 (6)	C19—C20	1.406 (8)
O1—C16	1.459 (6)	C19—H19	0.9500
O2—C30	1.376 (6)	C20—C21	1.397 (9)
O2—C33	1.449 (6)	C20—H20	0.9500
C1—C2	1.405 (8)	C21—C22	1.382 (7)
C1—C6	1.425 (8)	C22—C23	1.415 (7)
C2—C3	1.363 (8)	C22—H22	0.9500
C2—H2	0.9500	C23—C24	1.491 (7)
C3—C4	1.421 (8)	C24—C27	1.526 (7)
C3—H3	0.9500	C24—C25	1.532 (8)
C4—C5	1.398 (7)	C24—C26	1.557 (7)
C5—C6	1.391 (8)	C25—C26	1.479 (8)
C5—H5	0.9500	C26—H26A	0.9900
C6—C7	1.519 (7)	C26—H26B	0.9900
C7—C10	1.503 (7)	C27—C32	1.390 (7)
C7—C8	1.541 (8)	C27—C28	1.399 (7)
C7—C9	1.548 (8)	C28—C29	1.372 (7)
C8—C9	1.473 (8)	C28—H28	0.9500
C9—H9A	0.9900	C29—C30	1.400 (7)
C9—H9B	0.9900	C29—H29	0.9500
C10—C15	1.412 (7)	C30—C31	1.402 (7)
C10—C11	1.416 (7)	C31—C32	1.413 (7)
C11—C12	1.384 (8)	C31—H31	0.9500
C11—H11	0.9500	C32—H32	0.9500
C12—C13	1.401 (7)	C33—C34	1.511 (8)
C12—H12	0.9500	C33—H33A	0.9900
C13—C14	1.413 (7)	C33—H33B	0.9900
C14—C15	1.400 (8)	C34—H34A	0.9800
C14—H14	0.9500	C34—H34B	0.9800
C15—H15	0.9500	C34—H34C	0.9800
			
C13—O1—C16	118.7 (4)	H17B—C17—H17C	109.5
C30—O2—C33	117.5 (4)	C19—C18—C23	122.6 (5)
C2—C1—C6	120.5 (5)	C19—C18—Cl4	116.1 (4)
C2—C1—Cl1	118.6 (4)	C23—C18—Cl4	121.3 (4)
C6—C1—Cl1	120.8 (5)	C18—C19—C20	119.6 (5)
C3—C2—C1	121.0 (5)	C18—C19—H19	120.2
C3—C2—H2	119.5	C20—C19—H19	120.2
C1—C2—H2	119.5	C21—C20—C19	118.3 (5)
C2—C3—C4	119.1 (5)	C21—C20—H20	120.8
C2—C3—H3	120.5	C19—C20—H20	120.8
C4—C3—H3	120.5	C22—C21—C20	121.8 (6)
C5—C4—C3	120.5 (5)	C22—C21—Br2	119.5 (5)
C5—C4—Br1	120.9 (4)	C20—C21—Br2	118.6 (4)
C3—C4—Br1	118.5 (4)	C21—C22—C23	121.2 (5)
C6—C5—C4	120.8 (5)	C21—C22—H22	119.4
C6—C5—H5	119.6	C23—C22—H22	119.4
C4—C5—H5	119.6	C18—C23—C22	116.5 (5)
C5—C6—C1	118.0 (5)	C18—C23—C24	124.0 (5)
C5—C6—C7	120.5 (5)	C22—C23—C24	119.3 (5)
C1—C6—C7	121.2 (5)	C23—C24—C27	112.9 (4)
C10—C7—C6	112.4 (5)	C23—C24—C25	118.8 (4)
C10—C7—C8	119.4 (5)	C27—C24—C25	118.4 (5)
C6—C7—C8	119.2 (5)	C23—C24—C26	119.6 (5)
C10—C7—C9	117.7 (5)	C27—C24—C26	119.3 (5)
C6—C7—C9	120.8 (5)	C25—C24—C26	57.2 (3)
C8—C7—C9	56.9 (4)	C26—C25—C24	62.2 (4)
C9—C8—C7	61.8 (4)	C26—C25—Cl6	120.5 (4)
C9—C8—Cl3	121.6 (4)	C24—C25—Cl6	120.5 (4)
C7—C8—Cl3	118.0 (4)	C26—C25—Cl5	117.2 (4)
C9—C8—Cl2	116.5 (4)	C24—C25—Cl5	118.2 (4)
C7—C8—Cl2	119.6 (4)	Cl6—C25—Cl5	110.7 (3)
Cl3—C8—Cl2	111.4 (3)	C25—C26—C24	60.6 (4)
C8—C9—C7	61.3 (4)	C25—C26—H26A	117.7
C8—C9—H9A	117.6	C24—C26—H26A	117.7
C7—C9—H9A	117.6	C25—C26—H26B	117.7
C8—C9—H9B	117.6	C24—C26—H26B	117.7
C7—C9—H9B	117.6	H26A—C26—H26B	114.8
H9A—C9—H9B	114.7	C32—C27—C28	118.9 (5)
C15—C10—C11	116.6 (5)	C32—C27—C24	120.5 (5)
C15—C10—C7	121.8 (5)	C28—C27—C24	120.5 (5)
C11—C10—C7	121.2 (5)	C29—C28—C27	121.0 (5)
C12—C11—C10	121.8 (5)	C29—C28—H28	119.5
C12—C11—H11	119.1	C27—C28—H28	119.5
C10—C11—H11	119.1	C28—C29—C30	120.6 (5)
C11—C12—C13	120.7 (5)	C28—C29—H29	119.7
C11—C12—H12	119.6	C30—C29—H29	119.7
C13—C12—H12	119.6	O2—C30—C29	116.2 (5)
O1—C13—C12	116.6 (5)	O2—C30—C31	124.3 (5)
O1—C13—C14	124.2 (5)	C29—C30—C31	119.6 (5)
C12—C13—C14	119.2 (5)	C30—C31—C32	119.0 (5)
C15—C14—C13	119.1 (5)	C30—C31—H31	120.5
C15—C14—H14	120.4	C32—C31—H31	120.5
C13—C14—H14	120.4	C27—C32—C31	120.8 (5)
C14—C15—C10	122.4 (5)	C27—C32—H32	119.6
C14—C15—H15	118.8	C31—C32—H32	119.6
C10—C15—H15	118.8	O2—C33—C34	106.8 (4)
O1—C16—C17	108.0 (5)	O2—C33—H33A	110.4
O1—C16—H16A	110.1	C34—C33—H33A	110.4
C17—C16—H16A	110.1	O2—C33—H33B	110.4
O1—C16—H16B	110.1	C34—C33—H33B	110.4
C17—C16—H16B	110.1	H33A—C33—H33B	108.6
H16A—C16—H16B	108.4	C33—C34—H34A	109.5
C16—C17—H17A	109.5	C33—C34—H34B	109.5
C16—C17—H17B	109.5	H34A—C34—H34B	109.5
H17A—C17—H17B	109.5	C33—C34—H34C	109.5
C16—C17—H17C	109.5	H34A—C34—H34C	109.5
H17A—C17—H17C	109.5	H34B—C34—H34C	109.5
			
C6—C1—C2—C3	3.2 (9)	C23—C18—C19—C20	−1.7 (8)
Cl1—C1—C2—C3	−175.8 (5)	Cl4—C18—C19—C20	175.6 (4)
C1—C2—C3—C4	−2.4 (9)	C18—C19—C20—C21	0.8 (8)
C2—C3—C4—C5	0.2 (8)	C19—C20—C21—C22	0.2 (8)
C2—C3—C4—Br1	178.6 (4)	C19—C20—C21—Br2	−177.8 (4)
C3—C4—C5—C6	1.1 (8)	C20—C21—C22—C23	−0.4 (8)
Br1—C4—C5—C6	−177.2 (4)	Br2—C21—C22—C23	177.6 (4)
C4—C5—C6—C1	−0.3 (8)	C19—C18—C23—C22	1.5 (7)
C4—C5—C6—C7	173.4 (5)	Cl4—C18—C23—C22	−175.7 (4)
C2—C1—C6—C5	−1.8 (8)	C19—C18—C23—C24	176.8 (5)
Cl1—C1—C6—C5	177.2 (4)	Cl4—C18—C23—C24	−0.4 (7)
C2—C1—C6—C7	−175.5 (5)	C21—C22—C23—C18	−0.4 (7)
Cl1—C1—C6—C7	3.6 (7)	C21—C22—C23—C24	−176.0 (5)
C5—C6—C7—C10	−85.9 (6)	C18—C23—C24—C27	−95.2 (6)
C1—C6—C7—C10	87.5 (6)	C22—C23—C24—C27	80.0 (6)
C5—C6—C7—C8	61.1 (7)	C18—C23—C24—C25	119.6 (6)
C1—C6—C7—C8	−125.4 (6)	C22—C23—C24—C25	−65.2 (6)
C5—C6—C7—C9	127.9 (6)	C18—C23—C24—C26	53.1 (7)
C1—C6—C7—C9	−58.7 (7)	C22—C23—C24—C26	−131.7 (5)
C10—C7—C8—C9	−105.7 (6)	C23—C24—C25—C26	−108.5 (6)
C6—C7—C8—C9	109.6 (6)	C27—C24—C25—C26	108.2 (6)
C10—C7—C8—Cl3	7.2 (7)	C23—C24—C25—Cl6	140.7 (4)
C6—C7—C8—Cl3	−137.5 (5)	C27—C24—C25—Cl6	−2.6 (7)
C9—C7—C8—Cl3	112.9 (5)	C26—C24—C25—Cl6	−110.9 (5)
C10—C7—C8—Cl2	148.2 (4)	C23—C24—C25—Cl5	−0.8 (7)
C6—C7—C8—Cl2	3.5 (7)	C27—C24—C25—Cl5	−144.1 (4)
C9—C7—C8—Cl2	−106.2 (5)	C26—C24—C25—Cl5	107.7 (5)
Cl3—C8—C9—C7	−107.2 (5)	Cl6—C25—C26—C24	110.8 (5)
Cl2—C8—C9—C7	111.0 (5)	Cl5—C25—C26—C24	−109.3 (5)
C10—C7—C9—C8	108.6 (5)	C23—C24—C26—C25	107.0 (5)
C6—C7—C9—C8	−106.9 (6)	C27—C24—C26—C25	−106.7 (5)
C6—C7—C10—C15	−104.4 (6)	C23—C24—C27—C32	−78.0 (6)
C8—C7—C10—C15	108.6 (6)	C25—C24—C27—C32	67.4 (7)
C9—C7—C10—C15	42.9 (7)	C26—C24—C27—C32	133.6 (5)
C6—C7—C10—C11	68.8 (7)	C23—C24—C27—C28	98.1 (6)
C8—C7—C10—C11	−78.1 (7)	C25—C24—C27—C28	−116.6 (6)
C9—C7—C10—C11	−143.8 (5)	C26—C24—C27—C28	−50.3 (7)
C15—C10—C11—C12	0.8 (8)	C32—C27—C28—C29	0.4 (8)
C7—C10—C11—C12	−172.7 (5)	C24—C27—C28—C29	−175.7 (5)
C10—C11—C12—C13	0.2 (8)	C27—C28—C29—C30	−0.8 (8)
C16—O1—C13—C12	−172.7 (5)	C33—O2—C30—C29	−177.1 (5)
C16—O1—C13—C14	6.1 (8)	C33—O2—C30—C31	2.5 (7)
C11—C12—C13—O1	176.2 (5)	C28—C29—C30—O2	−179.6 (5)
C11—C12—C13—C14	−2.6 (8)	C28—C29—C30—C31	0.7 (8)
O1—C13—C14—C15	−174.9 (5)	O2—C30—C31—C32	−179.8 (5)
C12—C13—C14—C15	3.9 (8)	C29—C30—C31—C32	−0.2 (8)
C13—C14—C15—C10	−2.9 (8)	C28—C27—C32—C31	0.2 (8)
C11—C10—C15—C14	0.5 (8)	C24—C27—C32—C31	176.3 (5)
C7—C10—C15—C14	174.0 (5)	C30—C31—C32—C27	−0.3 (8)
C13—O1—C16—C17	173.7 (5)	C30—O2—C33—C34	179.3 (4)

### Hydrogen-bond geometry (Å, º)

Cg1 is the centroid of the C10–C15 ring. Cg2 is the centroid of the C27–C32 ring.

**Table:**

*D*—H···*A*	*D*—H	H···*A*	*D*···*A*	*D*—H···*A*
C3—H3···*Cg*1^i^	0.95	2.66	3.665 (3)	161
C20—H20···*Cg*2^ii^	0.95	2.60	3.480 (9)	155

Symmetry codes: (i) *x*, *y*−1, *z*; (ii) *x*, *y*+1, *z*.
